# Cognitive Difficulties Among Immigrant Latinos: Within Group Variation Across U.S. States

**DOI:** 10.1093/geroni/igaf122.1867

**Published:** 2025-12-31

**Authors:** Catherine Garcia, Marc Garcia

**Affiliations:** Syracuse University, Syracuse, New York, United States; Syracuse University, Syracuse, New York, United States

## Abstract

This study examined how U.S. state contexts shape disparities in cognitive disability risk among immigrant Latino adults, highlighting geographic variation in cognitive functioning. Using data from the 2008–2019 American Community Survey (ACS), we estimated a series of binary logistic regression models with clustered standard errors to assess differences in self-reported cognitive difficulties across (1) all Latinos by state, (2) immigrant Latinos by state, and (3) immigrant Latinos by country of origin and state. The analysis adjusted for gender, age, age², educational attainment, poverty status, and survey year. The sample included 901,016 self-identified immigrant Latinos aged 45 and older residing in one of 24 U.S. states that collectively account for 95% of the U.S. Latino population. Findings indicate substantial state-level variation in cognitive difficulties among foreign-born Latinos. Specifically, the probability of reporting cognitive difficulties was significantly higher among foreign-born Latinos residing in Northeastern states compared to other regions. Additionally, island-born Puerto Ricans exhibited the highest prevalence of cognitive difficulties relative to other Latino heritage groups across states. These results demonstrate how geographic contexts contribute to disparities in cognitive health among aging immigrant Latino populations. The observed variation underscores the role of structural and contextual factors in shaping cognitive disability risks, emphasizing the need to account for state-level influences when addressing Latino health inequities.

